# Exploring child and family-level influences on oral hygiene practices (OHP)—a qualitative study from South India

**DOI:** 10.22514/jocpd.2025.075

**Published:** 2025-07-03

**Authors:** Muthu Murugan, Vineet Dhar, Latha Nirmal, Ankita M Saikia, Shuba Kumar, Rani Mohanraj, Umapathy Pasupathy, Kalpana Balakrishnan

**Affiliations:** 1 Centre for Early Childhood Caries Research (CECCRe), Sri Ramachandra Dental College and Hospital, 600116 Chennai, India; 2 Department of Orthodontics and Paediatric Dentistry, University of Maryland School of Dentistry, Baltimore, MD 21201, USA; 3 Department of Public Health Dentistry, Sri Ramachandra Dental College and Hospital, 600116 Chennai, India; 4 Department of Paediatric Dentistry, UWA Dental School, 6009 Perth, WA, Australia; 5 Samarth, 600004 Chennai, India; 6 Department of Paediatrics, Sri Ramachandra Medical College and Research Institute, 600116 Chennai, India; 7 Department of Environmental Health Engineering, Sri Ramachandra Institute of Higher Education and Research, 600116 Chennai, India

**Keywords:** Early childhood caries, Parental perceptions, Oral hygiene practices, Qualitative study

## Abstract

Early childhood caries (ECC) is an oral health concern prevalent globally, and can be effectively prevented via improved oral health literacy and practices. Parental beliefs, attitudes and cultural factors widely influence children’s adoption of oral hygiene practices (OHP). Recent literature has demonstrated a knowledge gap in parents and caregivers regarding these practices. This qualitative study explored the traditional oral health beliefs, perspectives and practices among parents and caregivers of children aged 0–3 years, both with and without ECC via 24 semi-structured interviews and 2 focus group discussions. Moreover, information was collected regarding oral health practices and oral health-seeking behaviours to obtain in-depth insights into this phenomenon. The research process was guided by the Fisher-Owen’s model focusing on child- and family-level influences. The audio recordings were entered into the NVivo software and analysed using the thematic analytical approach. The analysis unveiled two main themes under child-level and three under family-level influences. The findings revealed that child- and family-level determinants, such as the tender age of the child and poor cooperation in complying with OHP coupled with poor parental awareness of the importance of oral hygiene, specifically tooth brushing methods, use of toothpaste, the role of fluoride in the prevention of ECC, nighttime brushing and poor self-efficacy contributed to its inadequate practice. Preventing ECC requires an attitudinal and behavioural change among parents and caregivers. These observations indicate the lack of knowledge about oral health and OHP amongst parents and underscore the need for enhanced parental awareness and understanding of OHP via educational programs, and the development of effective oral health policies to improve oral health outcomes in young children.

## 1. Introduction

Early childhood caries (ECC) refers to dental caries, typically affecting the primary dentition of infants and young children. ECC was re-defined as the presence of a primary tooth with one or more carious (non-cavitated or cavitated lesions), missing (due to caries) or filled surfaces in children under six years of age [1]. The sequelae of ECC have been reported to range from impaired growth and development, nutritional deficiencies and poor quality of oral health to suboptimal school attendance and performance [2]. These issues create societal pressures and strain financial resources [3]. There has been minimal improvement in oral health over the past two decades with approximately 573 million children with untreated dental caries worldwide [4]. In India the pooled prevalence of ECC is estimated to be 46.9% [5].

There is compelling evidence that parents’ attitudes, beliefs and perceptions of oral health, diet, oral hygiene practices (OHP) and regular visits to the dentist have a profound impact on children’s dietary habits and lifestyle behaviours [6]. In addition, parents residing in rural areas have knowledge gaps in oral health, which should be addressed [7]. Parents and other family members caring for pre-school children have limited awareness of oral health practices and their clinical implications and these practices may differ based on one’s culture and beliefs [8, 9]. Given the dependence of young children on their parents for the preservation and maintenance of good oral health, it is important to understand parental attitudes, practices, and belief systems regarding their children’s oral health [10]. Capturing and uncovering these parental perspectives via qualitative research methods can enhance our understanding of family-level influences that often guide these behaviours, thereby, enabling the development of appropriate educational messages related to the maintenance of good oral health and recognition and/or prevention of ECC [11].

This study aimed to explore the child- and family-level influences on OHP in young children. It is part of an ongoing research project that seeks to develop a mobile-based application for parents of children under 3 years of age, educating them about oral health, hygiene practices, and prevention of ECC in their children. As a first step, qualitative interviews and focus group discussions were conducted with such parents to understand their perceptions and beliefs regarding four domains of anticipatory guidance: oral development, OHP, diet and fluoride adequacy of their child. The information collected will, in turn, guide the development of the application by aiding in the creation of suitable and relevant content.

**FIGURE 1.**
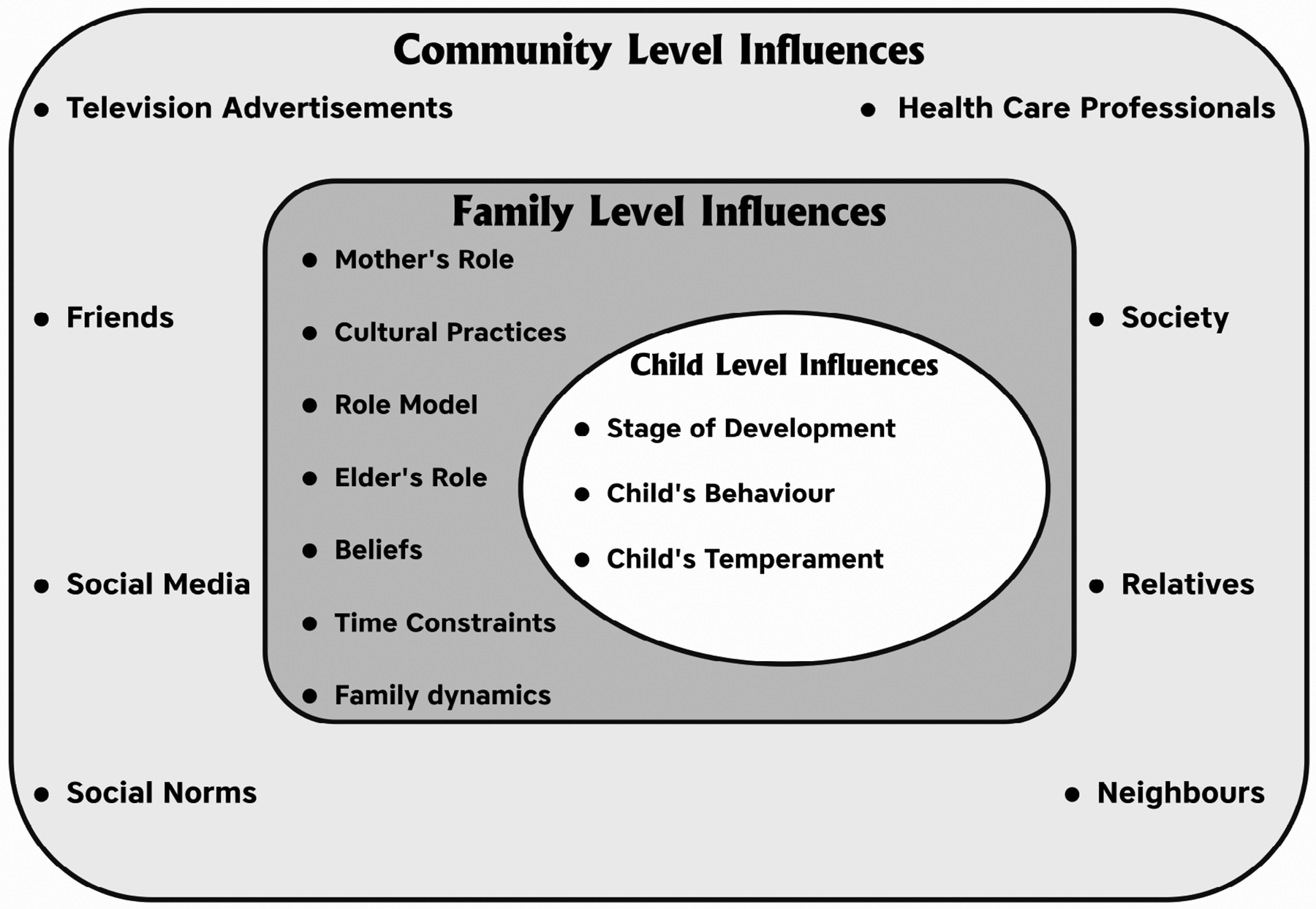
The Fisher-Owens model of parental insights on factors influencing oral hygiene practices.

Two theoretical models guided the overall conduct of the study, namely the Fisher-Owen’s model of child oral health and the Theory of Planned Behaviour [12, 13]. According to the Fisher-Owens model, genetic and biological factors, social environment, physical environment, medical and dental care systems and health behaviours operating at the child, family and community levels play a crucial role in children’s oral health and their oral health-seeking behaviours. These determinants must be considered early on to prevent ECC. Preventing ECC requires attitudinal and behavioural changes among caregivers [14]. This paper represents the foundational research undertaken to inform and aid in developing the intervention content. The Fisher-Owens model, which highlights the child and family-level influences on OHP, was used as a guiding framework for this study (Fig. 1).

## 2. Subjects and methods

COnsolidated criteria for REporting Qualitative research (CORE-Q) were followed for reporting this paper.

### 2.1 Study design and setting

This exploratory study utilised semi-structured interviews (SSIs) and focus group discussions (FGDs) to understand parents’ beliefs, perceptions, and attitudes regarding their children’s oral health. In addition, the norms and traditions around oral health practices and oral health-seeking behaviours were examined to obtain more profound insights into this phenomenon. This study was conducted at the Department of Paediatric Medicine and the Centre for Early Childhood Caries Research (CECCRe), SRIHER. Sri Ramachandra Medical Centre offers accessible, comprehensive, compassionate healthcare to patients from diverse socio-economic backgrounds.

### 2.2 Recruitment/Sampling

Parents of children (with and without ECC) <3 years of age visiting the Department of Paediatric Medicine and CECCRe, SRIHER who were willing to participate in the SSIs and FGDs were considered eligible for inclusion. Parents of children who were >3 years of age, very sick or previously treated for ECC and those unwilling to participate were excluded from the study.

### 2.3 SSIs

Using purposive sampling, parents who visited the outpatient departments (OPD) were approached and informed about the study. The parents were visiting the hospital for various reasons, including consultations, routine check-ups, vaccinations and treatments for various ailments in their children. The oral cavity of children aged 0–3 years was examined by a dentist and a research team member (LN) after prior permission from parents. The oral examination followed essential protocols to confirm the presence or absence of ECC. A total of 24 consenting parents were selected, which comprised 12 parents with children affected by ECC and an equal number of parents with children not affected by ECC. Parents of children in different age groups were selected to ensure diverse perceptions. Six age group clusters were identified: (i) 0–6 months, (ii) 7–12 months, (iii) 13–18 months, (iv) 19–24 months, (v) 25–30 months and (vi) 31–36 months. Two participants were recruited from each cluster: one from the ECC and one from the non-ECC group. Four parents of infants aged 0–6 months with no erupted teeth were also included. The interviews were conducted on the day of their visit to the OPD.

### 2.4 FGDs

To recruit participants to the FGDs, eligible parents who visited the OPDs were informed about the study’s purpose by research team member (LN) 2 weeks before the scheduled dates. The parents’ contact details and addresses were recorded after obtaining verbal consent and their willingness to participate in the planned FGD. Two FGDs were conducted on the hospital premises: one with 6 parents of children with ECC and one with 8 parents of children without ECC. Two experts, a psychologist (RM) and a social scientist (SK) with extensive experience in qualitative research methods, facilitated the SSIs and FGDs.

Given that the focus of our study was to develop an application-based education material on oral health and hygiene practices for parents of children <3 years of age towards the prevention of ECC, we believed these numbers were adequate to achieve saturation.

### 2.5 Guides for the SSIs and FGDs

A guide was prepared to aid in systematically collecting data. The guide included questions documenting the socio-demographic details of the parent and child, child-level determinants on development, physical attributes, family-level influences on social, cultural and family health behaviours. Furthermore, the guide contained questions regarding parental awareness and understanding of oral health, perceived behavioural controls, dietary habits and sleep-time feeding practices. Parental perceptions of poor oral health and its relationship to the child’s general health, as well as preventive measures, and factors that enable access to oral health care were also explored. Probes followed each question in the guide to encourage further exploration and understanding.

### 2.6 Data collection

The SSIs and FGDs were conducted privately within the hospital premises. The participants were provided with a comprehensive written consent document outlining the purpose of the SSI/FGD, and their willingness to participate was obtained. Moreover, permission to audio record the interviews/FGDs was obtained. Demographic information such as age, sex, educational background and geographic location (urban/rural), was gathered from all participants. Every effort was made to ensure that the primary purpose of the participant’s OPD visit was addressed before the SSIs and FGDs started.

The SSIs were conducted from 09 February to 13 March 2024. Each SSI took approximately 40–60 minutes to complete. A total of 24 SSIs were conducted, all in the regional language, Tamil. On days when both experts were available, a maximum of 3–4 participants were interviewed. As a token of appreciation for their participation, parents received a baby toothbrush, finger brush, or toy after the interviews. In addition, two FGDs were conducted in Tamil on the hospital premises, each lasting approximately 1 hour. All FGD participants received transportation reimbursement of INR 500 (approximately USD 6.00), a baby gift box (which included massage oil, baby soap, shampoo, and lotion) and a packed lunch after the session. Parents who had participated in the SSI were not included in the FGDs, and *vice versa*. None of the SSIs or FGDs were repeated.

### 2.7 Data analysis

The audio recordings of all 24 SSIs and the 2 FGDs were transcribed verbatim and translated into English. The transcripts were then imported into NVivo, Release 1.7.2 (QSR International Pty Ltd, Burlington, MA, USA) for coding the data and extracting the categories using the thematic analytical approach by the two qualitative research experts [15]. A hybrid deductive-inductive strategy was employed for data analysis. The Fisher-Owens model, which guided the study, constituted the deductive approach. This was followed by open coding of the data transcripts, which represented the inductive approach. A codebook was developed, encompassing codes and categories reflecting the study’s conceptual constructs. Following data collection, an inductive approach was applied to code the data, which aligned with the six principles of thematic analysis. This process commenced with data familiarisation via repeated readings of the transcripts, followed by the coding of each transcript. Both coders (RM and SK) divided all 26 transcripts (from 24 SSIs and 2 FGDs) and independently coded them using the established codebook. Subsequently, the codebook was expanded by adding the codes that were inductively derived from the interviews. The datasets from the coders were merged, and the codebook was reviewed. Any discrepancies between the coders were discussed and resolved, which led to the consolidation of the codebook. The third stage involved examining the coded data to verify the extent of data elucidation within the Fisher-Owens model context. The fourth stage entailed formalising the themes and assessing them for validity and credibility. Each theme was defined, labelled, and described in the fifth stage. Finally, a coherent explanation for the study findings was developed in the sixth stage of the analytical process. The data were sifted and organized, and the selected quotes were categorised under appropriate themes.

## 3. Results

### 3.1 Demographic characteristics

The demographic characteristics of 38 parents who participated in the study are summarised in Table 1. Thirty-two mothers predominantly identified as homemakers and aged 22–38 years, participated in the study. More than half of the parents had male children (22 out of 38), and over two-thirds of the participants lived in joint families (28 out of 38).

### 3.2 Themes of the analysis

**FIGURE 2.**
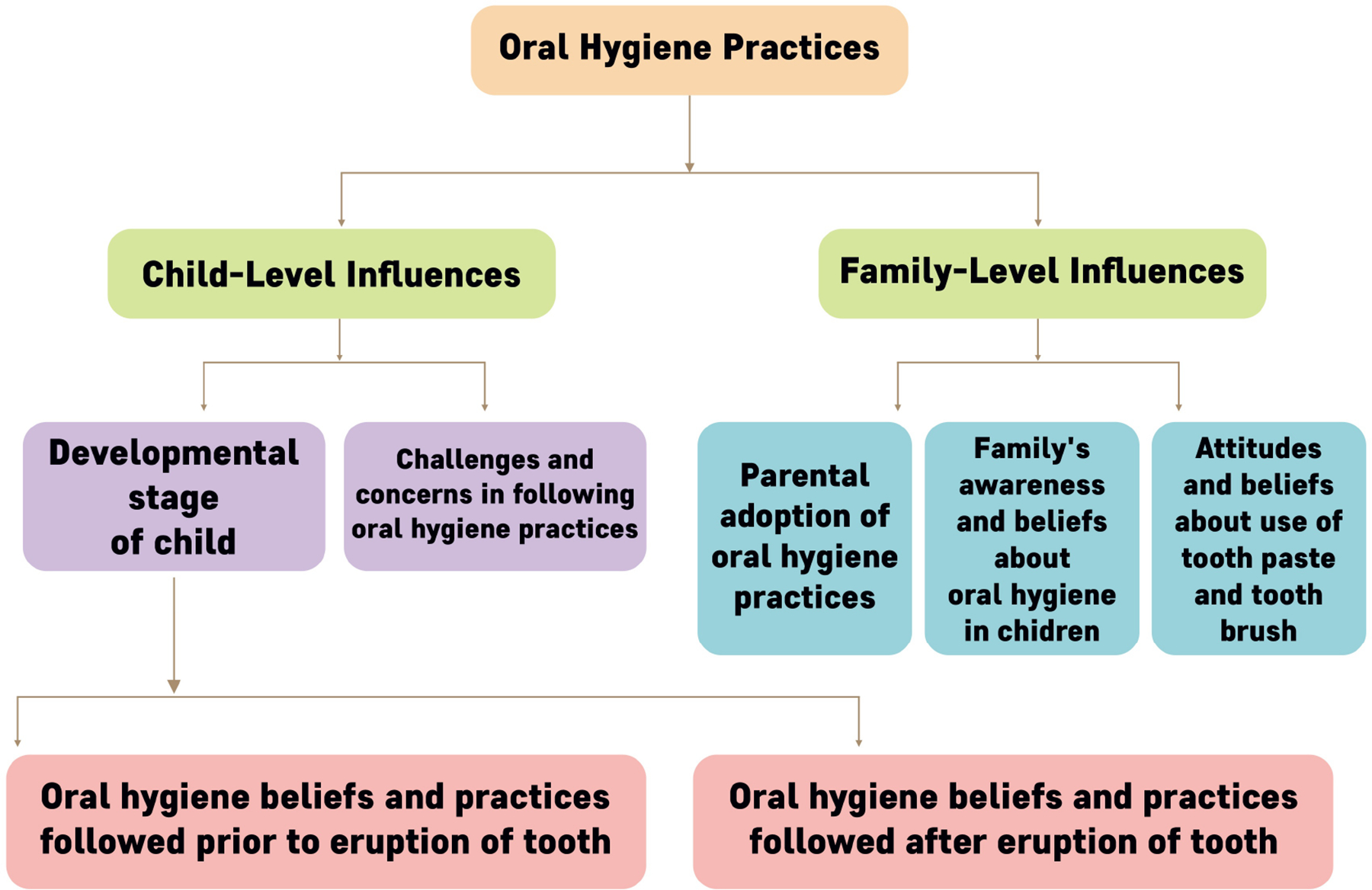
Themes of the analysis.

The central themes and underlying relationships identified under the child- and family-level influences are depicted in Fig. 2. The findings on child-level influences were presented under two themes: (i) the development stages of the child and (ii) the challenges and concerns related to following OHP. The first theme on the development stages further divided into two sub-themes: oral hygiene beliefs and practices before the eruption of teeth and those after the eruption. Under family-level influences, the findings were presented under three themes: parental adoption of OHP, family’s awareness and beliefs about oral hygiene in children, and attitudes and beliefs about using toothpaste and toothbrushes.

### 3.3 Child-level influences

#### 3.3.1 Developmental stage of child

##### 3.3.1.1 Oral hygiene beliefs and practices followed before the eruption of teeth

Most participants reported that their child’s first tooth erupted after the age of 6 months. The child was exclusively breastfed during the initial period, with minimal OHP. The participants did not perceive the need to clean the child’s gums or tongue.

“Until 3 months I will only wipe her lips after breastfeeding her, but her grandmother used to bathe the baby and she used to clean the mouth and gums.”

(ID 18, no caries, male, 27 months)

Most mothers reported feeding their child every 2 hours, with many children falling asleep while being breastfed or bottle-fed. There was no practice of cleaning the tongue or gums, nor rinsing the mouth with water after nighttime feedings as parents feared that these actions might awaken the child. A few mothers mentioned wiping their child’s lips after feeding. Some mothers reported feeling too tired at the end of the day to perform this practice. Almost all mothers reported cleaning the gums and tongue during the child’s bath time, using either their fingers or a soft cloth.

“We just wipe the mouth alone. We are scared that while they sleep, if we do something like cleaning, they will get disturbed, and if they wake up from sleep, what do we do?”

(FGD parents, no caries)

“I used to give a bottle of milk at night at 3.00 O’clock. I used to keep the milk in the flask. I will give that… I will not clean at that time… While he sleeps, we are not able to clean his mouth.”

(ID 15, no caries, male, 18 months)

“In the daytime 2 hours once she used to cry for milk. So, during nighttime, I myself wake up and give her milk 2 hours once. If it is enough, she won’t take milk. I don’t do specific cleaning for her mouth. When I give her a bath, I just clean her mouth with my finger. That’s it. No one said that it is important to clean the mouth every time.”

(ID 1, no caries, female, 6 months)

##### 3.3.1.2 Oral hygiene beliefs and practices after the eruption of teeth

With the introduction of solid foods coinciding with the eruption of multiple teeth, most participants engaged in OHP to certain extent. Providing water to drink after feeding was considered “sufficient enough” to clean the mouth. Some participants mentioned rinsing their child’s mouth with water after consuming solid foods. Many participants believed that cleaning the lips, mouth, and tongue during a child’s bath was “sufficient” following the introduction of solid foods. A few participants used their fingers to clean their children’s mouths when they had 4–8 erupted teeth. As more teeth erupted, some participants transitioned to using finger brushes. Most participants believed that providing water before meals and after snacks was a vital practice to maintain oral hygiene.

“When there were two teeth, I used to clean the teeth with my finger only. After 4–5 teeth, I used a brush to clean it……because of many teeth, if he eats anything it will be there in his teeth. So, I used a brush to clean that. I started using a finger brush when he was 9 months old. I wash his mouth every time he has food.”

(ID 19, no caries, male, 12 months)

“When he wakes up in the morning, I used to give water. That’s it; other than that, I won’t clean his teeth. I thought we would do it after more teeth came. Now he only has four teeth, and if we use the brush to clean them and if it scratches and she bleeds what do we do?”

(ID 10, no caries, male, 9 months).

A few participants spoke about the importance of maintaining proper hygiene for milk teeth, despite them being temporary. They believed that improper care of these teeth would negatively affect the growth of the permanent teeth. Some participants shared personal experiences of seeking dental care for tooth problems they had encountered or witnessed in family members. Through these experiences, they gained a deep understanding of oral hygiene.

“At the age of 3 or 5, it will fall down and another tooth will grow. After the age of 3 only it starts to fall…However, we have to keep our teeth clean. With that only we are able to eat. After we eat, something will stick in the teeth, and it will get affected.”

(ID 23, caries, male, 27 months)

“That first tooth will fall down at the age of 7 years only. Till then, we have to take care of it. When I came last time, they said that if the tooth root is affected new teeth also get affected. So, I have to take care and clean the teeth from the beginning itself.”

(ID 9, caries, male, 15 months)

#### 3.3.2 Challenges and concerns in following OHP

Several participants reported difficulties in making their child/children brush their teeth or cooperate when they attempted to brush their teeth for them, primarily because of the child’s developmental stage and age. In addition, the elders in the family often discouraged parents from brushing their children’s teeth, citing concerns about causing distress to the child and the risk of the child ingesting the toothpaste. Most participants indicated that the child’s cooperation and temperament were crucial factors that influenced their ability to adopt and practise oral hygiene routines. A few parents of children with dental caries said that their children resisted having their teeth brushed and complained of pain or sensitivity. In some cases, parents noted that their children preferred to be left alone and did not want to be supervised when brushing their teeth. Some parents believed that nighttime brushing was unnecessary for such young children. In contrast, although aware of its importance, others did not practise it because of fatigue at the end of the day or because it had a low priority compared with other competing demands. Parents from both groups exhibited a poor understanding of toothbrushing techniques, the recommended brushing duration, and the appropriate amount of toothpaste for their child.

“Brushing the teeth for the child is a challenge. They won’t cooperate. They close their mouth tightly and they won’t open it. Or they bite their teeth and sometimes they chew the brush itself. If we insert the brush in their mouth, it is difficult to take it out of their mouth. They hold the brush in their mouth.”

(Parents FGD, caries)

“Even now he is not allowing me to brush his teeth. Because of having small teeth, while using a brush, it is painful for him. So, he is not allowing me to use the brush at all. He is not saying that it is painful. He just won’t show it to me. But he will play with that brush by keeping it in his mouth. But he won’t allow me to brush.”

(ID 11, caries, male, 28 months)

“I started to use the brush when he was 17 months old. So, now I only thought to brush his teeth. But he is not allowing me to do it. He is shouting. So, we thought he himself would do what he wants to do. We left it as it is. After 1 year completed I myself started to give him a bath. When I give a head bath, I will clean his teeth with my finger.”

(ID 22, caries, male, 17 months)

“At the age of 1 year and 9 months, I started to brush her teeth. While brushing, she will start to cry, and she will swallow the paste. So I stopped using the paste…: Elders in the family told me that I can brush her teeth after she is a little older because she does not know to spit out. So, I too don’t use it.”

(Parents FGD, caries)

### 3.4 Family-level influences

#### 3.4.1 Parental adoption of OHP

Most participants reported brushing their teeth once a day. A few participants remarked that they brushed twice daily, gargled with water after every meal, particularly after experiencing caries in their youth, and understood the significance of maintaining good oral hygiene. Some participants did not appreciate the importance of brushing “twice-a-day” and admitted that they did not rinse their mouths after each meal. Although some participants were aware of the importance of “twice-a-day” brushing, they were inconsistent in their practices. Fatigue at the end of the day, concerns about enamel wear from frequent brushing, and a strong belief that nighttime brushing was unnecessary were some of the factors that dissuaded them from doing so. Moreover, some participants believed that brushing for an extended period in the morning compensated for not brushing at night. In addition, a few participants reported brushing their teeth at night, only if they had consumed sweet/sugary and/or non-vegetarian foods. One participant stated that although she practised brushing twice daily her family members were not motivated to do the same. Most participants said they gained information regarding OHP from various sources, including newspapers, television advertisements, the internet, social media, neighbours, family members, friends, relatives, colleagues and healthcare professionals.

“Today’s generations are all once in a day… no one in my family informed us… I have dinner at 10 PM. And I am too lazy to brush my teeth after that time.”

(ID 6, caries, male, 23 months)

“If I eat more non-veg food like fish, mutton and chicken and if it sticks in my teeth that time, I feel like brushing. Otherwise, if it is normal food, I don’t feel the need to brush my teeth.”

(ID 12, no caries, female, 3 months)

Some participants acknowledged that the practice of good oral hygiene should begin in childhood as it becomes established as a regular habit. Another participant mentioned that she and her mother-in-law brushed their teeth at night before dinner, similar to their morning routine before breakfast. Some participants agreed that the habit of brushing at night should be instilled and practised till it becomes a routine. A few participants, who had suffered from dental caries in their youth were more diligent in their OHP and passed this knowledge on to their children. Some admitted that their daily chores made them to forget or neglect to maintain healthy OHP. In contrast, others acknowledged that, they often failed to follow through despite their good intensions.

“I had that habit from childhood. After I eat at night I used to brush and go to sleep. Not brushing at night is unsettling for me. I have inculcated this practice in my children too. Because of that we don’t have any dental issues.”

(ID 13, no caries, male, 19 months)

“My brother and I used to brush twice in day. My mother and father would brush once a day, and they used to clean their mouth by gargling with water at night. My husband also brushes twice a day… I remember the doctor’s advice when I was studying 6th standard. We were following it from that day onwards. I used to do cleaning once in 6 months or 1 year. I go to a dental clinic. I had an issue and since it was like that, I thought I should give importance to it from now on and it should not damage other teeth. So, I am maintaining it.”

(ID 1, no caries, female, 6 months)

“When I was in my parent’s home my father taught me. When we wake up in the morning after brushing only, we drink tea or coffee. If we don’t brush, they won’t give us anything to eat at all. If we are too lazy to do the brushing they won’t give us food. My father brought us up like that. He taught us to brush twice.”

(ID 16, no caries, female, 24 months)

#### 3.4.2 Family’s awareness and beliefs about oral hygiene in children

There was a mixed picture regarding parents’ awareness and beliefs about the practice of oral hygiene in children. Some parents had no awareness and did not perceive it as necessary; others practised their own form of oral hygiene, and some understood and practised it diligently. For instance, some parents indicated being unaware of the need for or importance of nighttime brushing and rinsing the mouth after every feed. Others mentioned that the elders in their family dissuaded them from using the toothbrush or toothpaste or practising nighttime brushing for their child. They firmly believed that cleaning the child’s mouth with fingers alone was sufficient. One mother used coconut oil to clean her infant’s mouth, while another noted that her child had erupted teeth before 6 months of age but had refrained from using toothpaste or a toothbrush. The use of seed extracts to clean the infant’s tongue was also reported. Although most parents were aware that improper OHP and unhealthy dietary practices could lead to tooth decay, they remained erratic in their practices. Conversely, some parents understood and valued the practice of oral hygiene both for themselves and for their children. Most parents agreed that the elders in the family preferred that the mothers perform OHP.

“Coconut oil is used to clean the tongue…. After the naming function of the child, we started to clean his mouth and tongue with coconut oil. I will take oil in the finger and clean the tongue. If he sleeps at 9.00 PM…. he will wake up at 12.30 AM and after that early morning around 4.30 AM or 5.00 AM. I will give him milk when he wakes up at these times.”

(ID 13, no caries, male, 19 months)

“To wipe the tongue there is a separate seed. We just scrub it once on the floor, take it in a finger and wipe it on his tongue, which means it will take out all the coating on the tongue. It will clean his tongue. Once in a day only while he is taking a bath.”

(ID 23, caries, male, 27 months)

“If you brush your teeth clockwise up and down, the germs or dirt, come out from your teeth. So, I learnt it and I followed it, and I have an improvement in that. I thought about doing that for my child too. So, while brushing, I ask him to brush by rotation. When I do the brushing, I ask him to put a chair and stand on it, and I give him a brush to do it.”

(FGD, no caries)

Important to add, is that parents rarely ever asked any questions of their paediatricians regarding their child’s oral health and hygiene practices nor did they report their paediatricians offering any advice or recommendations on the matter. One mother reported feeling shy to seek information regarding her child’s teeth development from the doctor while another reported that doctors spent very little time explaining issues to them. The focus for most parents when they visited their paediatrician was on the child’s physical health with dental issues not a major concern.

“Doctors do not interact for longer time, only a handful of doctors interact more. Generally doctors are not explaining much.”

(ID 18, no caries, male, 27 months)

“So far, I didn’t ask anything to the doctor. I won’t talk much. I would like to ask more questions and clear my doubts, but I am shy to ask. Now you are talking to me, so I am asking everything to you. But everyone is not like that. If we go to the doctor because we have an issue he will write a medicine for that, and that is all.”

(ID 11, caries, male, 28 months)

“When we go to the paediatrician he does not check child’s teeth and all… he didn’t say anything at all about that… I also have not asked the paediatrician what brush and paste to use… When we bring our child to the hospital, we think about that cold should get cured, get cured from fever…. Let’s see other issue the next time.”

(ID 22, caries, male, 17 months)

#### 3.4.3 Attitudes and beliefs about the use of toothpaste and toothbrush

Most parents did not use toothpaste for children aged 0–36 months. Of those who did use toothpaste, its use was often delayed until the child was 18–24 months of age or during the pre-school years.

“I am not using toothpaste for my child…. Maybe some chemicals will be there, and it will cause infection to the child but 2 days once we make the child brush normally but not daily.”

(ID 17, no caries, female, 32 months)

Few participants were apprehensive about using toothpaste daily, fearing that the child might swallow it and ingest harmful chemicals. The participants randomly selected brands, most using toothpastes for adults such as Colgate and Pepsodent. Moreover, most participants were unaware of toothpastes specifically formulated for children.

“I have been using Colgate for a long time, and it is also good. It is good for the teeth, and it contains salt. So, I am using it for my children too.”

(ID 13, no caries, male, 19 months)

A few participants learnt about toothpastes specifically for children from advertisements and pharmacists in medical shops. One participant stated she chose green-coloured toothpaste as she presumed it contained fewer chemicals. Participants viewed ayurvedic toothpastes and toothpowders as being safe to swallow. Opinions on spitting and gargling varied among participants. Some believed that children could learn to spit and gargle by the 7th or 8th month, while most felt that children could not spit and, therefore, toothpaste should be avoided until the child is older. None of the parents reported consulting their paediatrician, regarding the choice of toothpaste.

“When she was younger, I used my finger to clean her mouth. My husband said that before the child is 1 year old, we should not use toothbrush and toothpaste to clean the child. After that, I used a brush with a little toothpaste to clean. After her first birthday I started to use a toothbrush and toothpaste to clean her mouth and teeth. Nowadays, I am asking her to gargle after eating anything because anything will stick in her teeth. So, gargle your mouth spit the water outside and don’t drink that water.”

(ID 20, Caries, Female, 33 months)

“Paediatrician didn’t tell anything about the teeth itself then how he will speak about paste. I didn’t feel like asking too. I watched the advertisement and felt the paste was ok…have seen another kid using it so for us different paste and for kids’ different paste.”

(ID 6, caries, male, 23 months)

Most parents were unaware of the benefits of “fluoride” in the toothpaste. Although 3 out of the 24 parents interviewed had heard the term, they were unaware of its role in preventing caries. There was a prevailing concern among parents that fluorides were poisonous.

“I heard about it (fluoride), but I don’t know that it should be in the toothpaste and that it would give strength to the gums. I have studied about it and know that, but I don’t know that we should check the toothpaste tto see if fluoride is there.”

(ID 15, no caries, male, 18 months)

“In the toothpaste there is a chemical called fluoride. If a man eats that fluoride, he will die. I am an engineer and basically, I know it. But, without fluoride, we cannot produce the paste at all. We take it daily. So, we are taking slow poison daily.”

(FGD, no caries)

Participants allowed their older children to use toothbrushes, but they were reluctant to do so for their younger children, fearing that the bristles might injure the soft gums and cause pain or infection. Several parents believed that toothbrushing for young children was unsafe and unnecessary. One mother stated that she initially used a toothbrush without toothpaste to initiate the tooth-brushing habit. A mother of two children observed that her younger son viewed his older brother as a role model, which encouraged him to adopt the habit earlier. After learning the correct way to brush her teeth from a dentist, one mother encouraged her son to brush his teeth alongside her so he could observe and learn the correct techniques. However, supervising the child while brushing was considered “time-consuming”, and a few mothers said they supervised their children’s brushing only once a week. A couple of mothers opined that they initially allowed their child to brush alone, and later, they brushed once more to ensure a germ-free mouth.

“I feel that the layer may wear off… because the paste has chemical content, it will affect that. And I have a feeling like that…I don’t know whether it is correct or not. But I feel like that…and because of that I feel one that one-time brushing is correct. I also plan to start using toothpaste for her when she is in the first standard.”

(ID 17, no caries, female, 32 months)

“We started brushing at the age of 2. But for him, he started at 1.5 years by seeing my elder kid. I learnt about the Colgate toothpaste for children by seeing the advertisement.”

(ID 6, caries, male, 23 months)

“We haven’t started brushing because full teeth have not appeared and the brush may poke, so she is not allowing us to brush her teeth since she is a small kid. But while we are brushing, she will use a small toothbrush and brush her teeth alone. After 1 year and 4 months more teeth started appearing and we used our fingers to clean them…. There were no teeth deep inside so I will give more importance once the teeth appear inside. I used to see using light if there were any cavities or not. When she is sleeping, I will open her mouth and see.”

(ID 21, no caries, female, 18 months)

“Even now he is not allowing me to keep a brush on his teeth. Because he has small teeth, using a brush is painful for him. So, he is not allowing me to use the brush at all. He is not saying that it is painful. He just won’t show it to me. But he will play with that brush by keeping it in his mouth. But he won’t allow me to do it.”

(ID 11, caries male, 28 months)

“When I do the brushing, I ask him to put a chair and stand on it, and I will give him a brush to use. Starting, I gave him an empty brush without tooth paste because he would swallow the paste and wouldn’t know how to spit. For 6 months I gave him a brush without toothpaste. After that he learnt to spit out. So, I kept a little paste and gave it to him for brushing.”

(FGD, no caries)

## 4. Discussion

This qualitative study presents the perspectives and beliefs of parents with children aged 0–3 years regarding OHP for their child. The study contributes to the existing literature by highlighting challenges and often overlooked viewpoints regarding OHP via a rigorous research design encompassing 24 SSIs and 2 FGDs. Using the Fisher-Owens model to explain the diverse child- and family-level determinants affecting children’s oral health outcomes further enhances the study’s rigour. Through this in-depth exploration, this investigation emphasises the need to create better awareness and understanding among parents/family members about the importance of OHP as a critical first step in promoting healthy oral behaviours in infants, toddlers and pre-schoolers.

ECC is linked to multiple biological and behavioural risk factors that can be easily prevented [16]. Nonetheless, it poses a substantial public health burden and contributes to the incidence of caries in permanent teeth [17]. Finlayson *et al*. [10] identified, that influencing factors at the child’s level are associated with their developmental stages, cooperation, resistance and temperament [10]. Participants in our study reported difficulties in maintaining a tooth brushing routine for their children, similar to the findings reported by Finlayson *et al*. [10] and Naidu *et al*. [18]. Increased resistance to brushing by young children has been documented in earlier studies [19, 20], where a child’s temperament has been related to a higher incidence of ECC [21]. Barriers to supervised brushing in older children included time constraints and the child’s attitude [22–24]. In our study, most participants with caries observed that their child resisted brushing due to sensitive teeth, gum bleeding, and pain, which have been corroborated in other studies [19]. Parents of children diagnosed with ECC were referred to CECCRe for management.

In our study, several mothers recognised their positive role in establishing healthy oral habits to prevent ECC in their children, often with minimal assistance from the other family members. Other studies have reported similar observations [19, 20]. Some mothers in our study frequently modelled tooth brushing to encourage their children and reinforce oral health routines, which agrees with the results of Suprabha *et al*. [20]. In addition, mothers and older siblings served as positive role models for younger children, motivating them to brush their teeth. Young children tended to imitate the brushing habits of older siblings and family members, which reinforced the practice of good oral hygiene, findings consistent with other studies [20, 25]. Nevertheless, many participants had an inadequate understanding of OHP, and they tended to give it a low priority. Therefore, fostering a positive appreciation of the importance of OHP, diet and dental care is imperative. Parents and families should be made to understand that the risk of caries increases in children when the practice of oral hygiene is poor or inconsistent or when parents have a limited comprehension of these concepts [26, 27].

Despite most mothers in our study having graduate-level education, family dynamics often placed the final decision-making authority with elderly members. Our findings revealed unique OHP, such as coconut oil to clean the mouth and using a seed extract to clean the tongue, among the participants. To the best of our knowledge, these practices have not been previously documented in scientific literature. Community-level influences, including information from television advertisements, social media, neighbours, friends, relatives and healthcare professionals, shaped parents’ awareness of oral health care and informed their choices regarding oral hygiene products, such as toothbrushes and fluoridated or non-fluoridated toothpaste. These results agree with those of Finlayson *et al*. [10] and Naidu *et al*. [18].

Although numerous investigations have examined parents’ perspectives on OHP for children <6 years of age, concrete evidence is lacking regarding hygiene practices following breastfeeding or bottle-feeding during the initial edentulous stage or the gum-pad stage. Our findings confirm that parents’ awareness of OHP during this period was limited. Our study contradicts the findings of Lamba *et al*. [28] which suggested that parents began cleaning their children’s mouths immediately after birth. Most participants in our study reported using their fingers to clean their child’s teeth during bathing. Our observations are aligned with the findings of Shaheen *et al*. [6] and Naidu *et al*. [18], who reported using finger brushes to clean >3 teeth. Participants indicated that water was the primary agent for oral cleaning, consistent with Naidu *et al*. [18]. Although most mothers recognised the importance of nighttime brushing, they struggled to implement it for their children because of various barriers, such as fatigue, time constraints, stress, lack of family cooperation, challenging child behaviour, children going to bed early, and busy schedules. These findings are corroborated by Suprabha *et al*. [20]. Factors such as poor awareness of correct brushing methods, lack of cooperation from the child, inability to manage the child’s behaviour, and pressure from family elders to avoid distressing the child were cited as common obstacles to practising good oral hygiene behaviours within families.

Most participants exhibited fragmented knowledge regarding age-appropriate brushing techniques and demonstrated a lack of self-efficacy [26]. A study by Wilson *et al*. [29] has asserted that mothers aware of their children’s oral health needs tended to feel more efficacious. However, a considerable knowledge gap prevailed regarding the type and amount of toothpaste appropriate for different age groups, introducing tooth brushing, age-appropriate toothbrushes, brushing duration and the role of fluorides in toothpaste. Similar findings have been reported by Suprabha *et al*. [20] and Chalvatzoglou *et al*. [26]. Most parents practised brushing once a day, and although they acknowledged the importance of twice-daily brushing, they did not consistently adhere to this practice, which contradicts the results of Naidu *et al*. [18].

The strength of this study lies in its qualitative design, which enables a profound analysis of the direct processes influencing the parents of children <3 years of age in adopting OHP. The interactive discussions and probing questions addressed the fundamental shortcomings of conventional questionnaire-based studies. This investigation is the first to obtain insights from participants regarding infant oral care practices. Using SSIs and FGDs improved the credibility of the findings by providing personal and normative data. Research rigour was ensured by meticulously documenting the procedures and data collection methods and using a hybrid deductive-inductive coding approach. Further, this study was guided by the Fisher-Owens model with the focus on child and family-level influences. During the interviews, each domain of the model was thoroughly explored and at the stage of coding we kept our minds open to the generation of new codes beyond the ones already developed which enabled saturation of each domain of the model.

The study was conducted in a healthcare facility (a tertiary hospital) where participants sought treatment for general health issues and dental concerns. Consequently, the interviews and discussions were held in the OPD, and participants could have faced inhibitions in freely expressing their views. The study’s transferability may be limited, as all participants were drawn from a single private tertiary facility in South India, where cultural and familial practices may differ from those in other regions of India.

## 5. Conclusions

Our study indicates that parents of children <3 years of age demonstrate limited awareness and understanding of oral health and hygiene practices. This finding highlights the need to identify opportunities to promote oral health education and develop policies to improve oral health outcomes for young children.

## Figures and Tables

**TABLE 1.** Participant’s characteristics (N = 38).

S. No.	Variables	Categories	N	%
1	Age of the child in months	0–6	4	10.5
		7–12	5	13.2
		13–18	4	10.5
		19–24	9	23.7
		25–30	11	28.9
		31–36	5	13.2
2	Gender of the child	Male	22	57.8
		Female	16	42.1
3	Order of the child	1	27	71.0
		2	10	26.3
		3	1	2.6
4	Number of siblings	None	20	52.6
		1	16	42.1
		2	1	2.6
		3	1	2.6
5	Participating parent/caregiver	Mother	32	84.2
		Father	5	13.1
		Grandmother	1	2.6
6	Mother’s age in years	21–25	12	31.5
		26–30	19	50.0
		31–35	6	15.7
		36–40	1	2.6
7	Father’s age in years	21–30	13	34.2
		31–40	24	63.1
		41–50	1	2.6
8	Family’s composition	Joint	28	73.6
		Nuclear	10	26.3
9	Mother’s education	University	27	71.1
		Higher secondary	5	13.1
		High school	6	15.7
10	Father’s education	University	20	52.6
		Higher secondary	6	15.7
		High School and others	12	31.5
11	Mother’s occupation	Home maker	32	84.2
		Working mothers	6	15.7
12	Father’s occupation	Engineer	15	39.4
		Banking	2	5.2
		Business	4	10.5
		Miscellaneous—carpenter, designer, farmer, drivers, conductor, ATM cash loader, chef, student, building worker, supervisor, Asst manager auto drivers	17	44.7

ATM: Automated Teller Machine; Asst: Assistant.

## Data Availability

Most of the relevant excerpts from the qualitative interviews are included in the manuscript.

## ABBREVIATIONS

ECC: early childhood caries
OHP: oral hygiene practices
CECCRe: Centre for Early Childhood Caries Research
SRIHER: Sri Ramachandra Institute of Higher Education and Research
OPD: outpatient department
SSI: semi-structured interview
FGD: focus group discussion
